# Verbal intelligence and leisure activities are associated with cognitive performance and resting-state electroencephalogram

**DOI:** 10.3389/fnagi.2022.921518

**Published:** 2022-10-04

**Authors:** Martina Ferrari-Díaz, Ricardo Iván Bravo-Chávez, Juan Silva-Pereyra, Thalía Fernández, Carmen García-Peña, Mario Rodríguez-Camacho

**Affiliations:** ^1^Facultad de Estudios Superiores Iztacala, Universidad Nacional Autónoma de México, Tlalnepantla, Mexico; ^2^Departamento de Neurobiología Conductual y Cognitiva, Instituto de Neurobiología, Universidad Nacional Autónoma de México, Juriquilla, Mexico; ^3^Departamento de Investigación, Instituto Nacional de Geriatría, Ciudad de México, Mexico

**Keywords:** cognitive reserve, resting-state EEG, cognition, dynamic proxies, healthy aging, verbal intelligence, leisure activities

## Abstract

Cognitive reserve (CR) is the adaptability of cognitive processes that helps to explain differences in the susceptibility of cognitive or daily functions to resist the onslaught of brain-related injury or the normal aging process. The underlying brain mechanisms of CR studied through electroencephalogram (EEG) are scarcely reported. To our knowledge, few studies have considered a combination of exclusively dynamic proxy measures of CR. We evaluated the association of CR with cognition and resting-state EEG in older adults using three of the most frequently used dynamic proxy measures of CR: verbal intelligence, leisure activities, and physical activities. Multiple linear regression analyses with the CR proxies as independent variables and cognitive performance and the absolute power (AP) on six resting-state EEG components (beta, alpha1, alpha2, gamma, theta, and delta) as outcomes were performed. Eighty-eight healthy older adults aged 60–77 (58 female) were selected from previous study data. Verbal intelligence was a significant positive predictor of perceptual organization, working memory, processing speed, executive functions, and central delta power. Leisure activities were a significant positive predictor of posterior alpha2 power. The dynamic proxy variables of CR are differently associated with cognitive performance and resting-state EEG. Implementing leisure activities and tasks to increase vocabulary may promote better cognitive performance through compensation or neural efficiency mechanisms.

## Introduction

The normal aging process brings several structural and functional brain changes with gains and losses in cognition (Fjell and Walhovd, 2010; Juraska and Lowry, 2012; Spreng and Turner, 2019). For instance, increasing age is associated with better performance in general knowledge and semantic information accumulated over the life course (i.e., crystallized intelligence), whereas fluid cognition, such as the ability to process information and solve problems, declines steadily (Park and Reuter-Lorenz, 2009; Harada et al., 2013; Spreng and Turner, 2019).

These changes in cognition seem to relate to changes in brain functioning (Fjell and Walhovd, 2010; Toepper, 2017). A technique to assess brain functioning is electroencephalography (EEG). Studies have shown that cognitive decline is associated with reduced power in the alpha frequency band (Roca-Stappung et al., 2012; Vlahou et al., 2014; Barry and De Blasio, 2017; Choi et al., 2019; Kamal et al., 2020; Kumral et al., 2020) and increased power in theta and delta frequency bands (Roca-Stappung et al., 2012; Vlahou et al., 2014; Barry and De Blasio, 2017; Choi et al., 2019; Kamal et al., 2020; Kumral et al., 2020), referred to as generalized EEG slowing, in the resting-state EEG of healthy older subjects (50 years or more). However, the interaction between neuropathology (e.g., amyloid load, tangle density, cerebral infarcts) and the level of cognitive function seems to be a non-linear relationship (Bennett et al., 2006, 2012). There is evidence of individuals who present pathological brain features of Alzheimer’s disease (AD) or the aging process, but they do not display major clinical symptoms of cognitive decline (Bennett et al., 2006, 2012; SantaCruz et al., 2011; Boyle et al., 2013; Castro-Chavira et al., 2016). This variation may be attributable to multiple factors, such as their level of education, occupational attainment, participation in leisure and physical activities, or social networks (Arenaza-Urquijo et al., 2015; Qiu and Fratiglioni, 2018).

Efforts have been made to investigate the factors that may reduce the impact of aging on both brain structure and function to better understand individual differences in cognitive abilities (Cabeza et al., 2018; Stern et al., 2019, 2020). An extensively studied concept that seems to alter the effect of age-related brain changes on cognitive performance is the cognitive reserve (CR). CR is the adaptability of cognitive processes that helps to explain differences in the susceptibility of cognitive or daily functions to resist the onslaught of brain-related injury or the normal aging process (Stern et al., 2020).

CR is a concept that challenges researchers to evaluate it (Stern, 2009). Frequently, CR has been studied by using proxy variables such as years of education and occupational attainment, attributes that remain static after mid-adulthood. Years (or level) of education is the most used proxy variable in studies of CR (Harrison et al., 2015; Opdebeeck et al., 2016). Dynamic proxy measures (modifiable factors) seem to more accurately reflect the influence of CR on cognition, as reported by Malek-Ahmadi et al. (2017), who compared years of education (static measure) and verbal intelligence (dynamic measure). One of the dynamic proxy measures most used in studies is verbal intelligence (Opdebeeck et al., 2016; Nogueira et al., 2022). Nevertheless, other dynamic proxy measures, such as leisure and physical activities, may contribute to a better understanding of CR plasticity on both cognitive and brain function (Malek-Ahmadi et al., 2017). Contrary to static proxy measures, leisure and physical activities can be voluntarily and easily implemented by individuals in later life, producing active changes in their routine, and are considered to be characteristic components of a healthy lifestyle (Wang et al., 2012; Fallahpour et al., 2016; Erickson et al., 2019).

The underlying mechanisms of CR seem to rely on the interaction of different brain networks (Stern et al., 2019, 2020). Cabeza et al. (2018) propose three mechanisms: reserve, a cumulative improvement in neural resources that mitigates brain injury or age-related decline; maintenance, the preservation of neural resources that implies a constant repair of the brain; or compensation, neural recruitment to enhance the performance of a high cognitive demanding task.

These brain mechanisms of CR have been scarcely studied through EEG even though it directly assesses neuronal processing. The few studies that explore the relationship between CR and EEG are heterogeneous in design, samples, and measures (Šneidere et al., 2020; Balart-Sánchez et al., 2021). Furthermore, the proportion of the studies decreases if we focus on resting-state EEG, which evaluates spontaneous and intrinsic neural activity independently of cognitive task demands (Fleck et al., 2017), and can inform us about the functional integrity of the brain (Harmony, 2009).

CR and resting-state EEG research have been shown to depend on the proxy measure used, either static or dynamic. A set of studies considered just one proxy measure of CR (dynamic or static), such as educational level (Babiloni et al., 2020) or incidental physical activity (Sanchez-Lopez et al., 2018). The remaining studies indistinctly use a combination of dynamic and static proxy measures of CR: a composite of verbal intelligence and education (Fleck et al., 2017), the total score of the Lifetime Experience Questionnaire (LEQ; Valenzuela and Sachdev, 2007) that assesses educational, leisure, social, and occupational history (Moezzi et al., 2019), or a composition through factorial analysis of proxy measures of CR named as a cognitive factor (education, IQ, and occupation), social factor (leisure and social activities), and exercise factor (physical activities and IQ; Fleck et al., 2019). The main findings of these studies on CR and resting-state EEG in the eyes-closed condition showed that a higher CR is associated with higher alpha power (Sanchez-Lopez et al., 2018; Babiloni et al., 2020), higher alpha and theta coherence (Fleck et al., 2017), higher alpha and theta lagged linear connectivity (LLC; Fleck et al., 2019), reduced theta power (Sanchez-Lopez et al., 2018), and reduced alpha imaginary coherence (Moezzi et al., 2019). In the eyes-open condition, a higher CR is related to higher alpha1 and theta LLC (Fleck et al., 2019) and higher theta imaginary coherence (Moezzi et al., 2019).

The results on cognition in these studies have shown that a higher CR is associated with higher scores in spatial working memory, sustained attention (Fleck et al., 2019), digit span, fluency (Fleck et al., 2017), matrix reasoning, digit-symbol coding, picture arrangement (Sanchez-Lopez et al., 2018), general cognition assessed by the Mini-Mental State Examination (MMSE; Fleck et al., 2017), performance IQ (Sanchez-Lopez et al., 2018), and Addenbrooke’s Cognitive Examination-Revised (ACE-R; Moezzi et al., 2019). Only one study controlled for general cognition (MMSE; Babiloni et al., 2020), and another study did not find an association between the exercise factor and EEG and cognition (Fleck et al., 2019). A meta-analysis reported positive correlations between different cognitive domains (language, memory, working memory, executive function, visuospatial abilities, and general cognition) and three proxy measures of CR (measured through education, occupational status, and engagement in cognitively stimulating activities) in healthy older adults (Opdebeeck et al., 2016).

Evidence suggests a relationship of CR with alpha and theta bands, yet the direction of this association is still ambiguous. One reason that could explain the heterogeneity of the results may be the different CR proxies employed. The usage of composite scores, scales, questionnaires (Cognitive Reserve Index questionnaire, LEQ, Valenzuela and Sachdev, 2007; CRIq, Nucci et al., 2012) or a combination of factors has been recommended to accurately assess CR variability (Harrison et al., 2015; Opdebeeck et al., 2016), but there is evidence of a different association with cognition and brain functioning between static and dynamic proxy measures of CR (Malek-Ahmadi et al., 2017; Serra et al., 2019). The first ones seem to relate to crystallized knowledge and differentiate hippocampal and parahippocampal volumes in AD patients; the second ones correlate more with fluid abilities and can distinguish individuals since the amnestic mild cognitive impairment (aMCI) stage (Malek-Ahmadi et al., 2017; Serra et al., 2019). Despite this evidence, few studies about resting-state EEG assessed CR as a composition of variables of just one category.

Therefore, the aim of our study was to evaluate the association of CR with cognition and resting-state EEG in healthy older adults using three of the most frequently used dynamic proxy measures of CR: verbal intelligence, leisure activities, and physical activities. To study brain electrical activity, we employed resting-state EEG, which has been widely used to assess cognitive and brain changes in healthy and pathological aging (Koenig et al., 2020) but has been scarcely used in CR studies (Šneidere et al., 2020; Balart-Sánchez et al., 2021).

We hypothesized that healthy older adults with higher dynamic CR would show better cognitive performance on fluid cognitive abilities (Serra et al., 2019), greater power in alpha (Fleck et al., 2017, 2019; Sanchez-Lopez et al., 2018; Babiloni et al., 2020), and reduced power in theta (Sanchez-Lopez et al., 2018) in resting-state EEG eyes-closed conditions compared to participants with lower dynamic CR.

## Materials and methods

### Design and sample

We conducted a secondary analysis of the study by Sanchez-Lopez et al. (2018). Complete objectives and procedures are available elsewhere (Sanchez-Lopez et al., 2018).

Participants were enrolled according to the following inclusion criteria: absence of cognitive decline symptoms considering the scores from the Global Deterioration Scale (GDS; Reisberg et al., 1982), the MMSE (Folstein et al., 1975), and the brief neuropsychological test battery in Spanish (NEUROPSI; Ostrosky-Solís et al., 1999); absence of depressive symptoms indirectly evaluated by the Quality of Life Enjoyment and Satisfaction Questionnaire (Q-LES-Q; Endicott et al., 1993); normal intellectual ability assessed by the Wechsler Adult Intelligence Scale in Spanish (WAIS-III-R; Wechsler, 2003); and absence of major socioeconomic disadvantages evaluated by The Mexican Association of Marketing Research and Public Opinion Agencies (The Mexican Association of Marketing Research and Public Opinion Agencies [AMAI] 8 x 7, 2018) questionnaire, because previous studies have demonstrated how socioeconomic deprivation influences cognitive performance and EEG (Wu et al., 2016; Maguire and Schneider, 2019; Zhang et al., 2022). Additionally, participants who at least completed junior high school were included to control the influence of this static proxy measure of CR. Volunteers were evaluated by a geriatric psychiatrist and were excluded from the study if they presented any psychiatric or neurological disorder; they were also excluded if they had abnormal levels of cells in a complete blood count, cholesterol, triglycerides, glucose, or thyroid-stimulating hormone.

The present study also considered the inclusion criteria of right-handedness, and we excluded participants acquired with a sampling rate of 100 Hz, which was insufficient to analyze the activity of the gamma frequency band.

For the present report, we included 88 healthy older adults (58 women, 30 men). Their ages ranged from 60 to 77 years.

All participants signed informed consent forms that were approved by The Ethical Committee of the Institute of Neurobiology at the National Autonomous University of Mexico (INEU/SA/CB/109, protocol 030-H-RM).

### Sociodemographic variables

The sociodemographic variables were assessed through a brief interview. Age was considered in years, and sex was a dichotomic variable (male = 1, female = 0). Education was categorized into four levels, starting from junior high school because of the inclusion criteria: (1) junior high school, (2) high school, (3) university/college graduate (first degree), and (4) postgraduate (master’s and doctoral degree).

### Dependent variables

#### Cognition

##### Wechsler adult intelligence scale in Spanish (*WAIS-III-R*)

This is used to assess the cognitive ability of adolescents and adults who are 16–90 years and 11 months old, and it is standardized for the Mexican population (Wechsler, 2003). This test consists of 13 subtests. From the score obtained for each of these subtests, three intelligence quotient (IQ) scores (verbal IQ, performance IQ, and full-scale IQ) and four index scores (verbal comprehension index, VCI; working memory index, WMI; perceptual organization index, POI; and processing speed index, PSI) were calculated. These indices are integrated by some of the 13 subtests: (a) VCI, similarities, vocabulary, information, and comprehension; (b) WMI, arithmetic, digit span, and letter-number sequencing; (c) POI, picture completion, block design, matrix reasoning, and picture arrangement; (d) PSI, symbol search, and coding.

For data analysis, we used the scalar score of the vocabulary subtest for CR and three index scores (WMI, POI, and PSI) for cognitive performance. VCI was excluded because the total score is also composed of the vocabulary subtest.

##### Brief neuropsychological test battery in Spanish (NEUROPSI)

This instrument assesses cognitive function in people from 16 to 85 years old and is standardized for the Mexican population (Ostrosky-Solís et al., 1999). It is particularly used on neurological, geriatric, and psychiatric patients. The battery is composed of different subtests that evaluate the following cognitive processes: (a) attention and concentration (digit span, visual detection, and subtraction), (b) memory (encoding and retrieval of a list of words and a semicomplex figure), (c) language (semantic and phonological fluency, denomination, repetition, and comprehension), and (d) executive and motor functions (motor programming and opposite reactions). We included semantic and phonological fluency in the executive functions as considered by some authors (Piatt et al., 1999; Snyder and Munakata, 2008).

For data analysis, we computed the standardized scores of each subtest into a composite score for every cognitive process: attention, memory, language, and executive functions.

#### Electroencephalogram

In a sound-proof, faradized, and dimly lit room, 12 (from 10 to 15) minutes on average of resting-state EEG at eyes-closed condition were recorded from each participant using the Medicid™ IV System (Neuronic Mexicana, S.A.; México) and EEG signal acquisition software (Track Walker™ v2.0). EEG data were recorded using 19 channels of the 10/20 system (ElectroCap™, International Inc.; Eaton, Ohio), referred to the linked earlobes (A1A2). The amplifier bandwidth was set between 0.50 and 50 Hz, and the sensor impedance levels were at or below 10 kΩ. Data were sampled at 200 Hz, and the EEG signal was amplified with a gain of 20,000. Participants were instructed not to take any sleeping pills the night before, to sleep at least 6 h and to go about their morning activities normally. EEG recordings were made between 8:00 and 12:00 in the morning. To rule out the presence of slow activity due to drowsiness, breaks were taken regularly.

Each participant’s EEG record was visually inspected offline for artifacts by an expert electroencephalographer. No participant showed paroxysmal activity. One criterion used in EEG editing was that the frequency or amplitude of the posterior rhythm should not be reduced with respect to what it had been at the beginning of the recording. Twenty-four artifact-free segments of 2.56 s were selected for quantitative analysis.

The preprocessing and quantitative EEG analyses were performed offline using EEGLAB (Delorme and Makeig, 2004) and a customized script in MATLAB software (The MathWorks Inc., Natick, MA, USA). The data were filtered from 0.5 to 50 Hz. Artifact subspace reconstruction, a method that eliminates high amplitude noise, including movement-related artifacts (Mullen et al., 2015), was performed. Absolute power (AP) was calculated by applying the fast Fourier transform for each electrode within different EEG frequency bands, including delta (0.5–3.5 Hz), theta (3.6–7.5 Hz), alpha1 (7.6–10 Hz), alpha2 (10.1–12.5 Hz), beta1 (12.6–16.5 Hz), beta2 (16.6–20.5 Hz), beta3 (20.6–30.5 Hz), and gamma (30.6–50 Hz).

Principal component analysis (PCA) with varimax rotation was then performed on the AP at the 19 electrodes in the eight frequency bands to reduce the dimension of the variable space. The PCA results showed 15 components using Kaiser’s criterion (Kaiser, 1960) that explained 92.67% of the total variance, but only the first 11 components that explained 88.11% of the total variance included all 152 variables (Supplementary Table 1). The factor scores of the first 11 components were calculated.

We selected the first six components that explained 72.49% of the total variance because they were primarily composed of electrodes from the following frequency bands: beta (12.6–30.5 Hz), alpha1, alpha2, gamma, theta, and delta (Supplementary Figure 1). The last five components were excluded because they were composed of just a few of the remaining electrodes of the delta, beta, or gamma frequency bands (Supplementary Table 1 and Supplementary Figure 1).

For data analysis, we used the factor scores of the first six components.

### Independent variable

#### Cognitive reserve

To assess CR, we considered three dynamic proxy measures: (a) verbal intelligence, using the score of the vocabulary subtest of WAIS-III-R (Wechsler, 2003); (b) leisure activities, using the total score of an adaptation from the “hobbies” dimension of the Cognitive Reserve Scale (CRS) pilot study (León et al., 2011, 2014); and (c) physical activity, considering the total index of the Yale Physical Activity Survey (YPAS; Dipietro et al., 1993; De Abajo et al., 2001). We selected verbal intelligence as the most frequently used dynamic proxy measure and leisure and physical activities as factors that account for the later life variability of CR.

##### Hobbies dimension

We elaborated questions from the items related to hobbies presented in the pilot study of the CRS (León et al., 2011, 2014): reading, playing games, writing, listening to music, watching TV, playing a musical instrument, collecting objects, traveling, attending cultural events, crafting, cooking, painting/taking pictures, shopping, and doing physical activity. We assessed the frequency of these activities with a Likert-type scale from 0 to 4 points into three different life stages (young adulthood, adulthood, and late adulthood). To account for the development of CR throughout their lifespan, we considered the total score of the three life stages of the participants.

For data analysis, we used the total score of the different activities from the three life stages.

##### Yale physical activity survey (YPAS)

The YPAS is a questionnaire integrating two sections that describe the everyday physical activation in older adults (Dipietro et al., 1993), and has been adapted into a Spanish version (De Abajo et al., 2001). The first part comprises different items about time spent (hours per week) on work, exercise, and recreational activities. The second part consists of five items with categorical options (frequency and time spent in minutes or hours) about different physical activities (vigorous activity, leisurely walking, moving, standing, and sitting). For each type of activity, an index is computed: the frequency and duration of each activity are multiplied by a weighting factor based on the intensity of the activity.

For data analysis, we used the final index (total physical index), which is the sum of the five indices of the second part.

### Statistical analysis

All statistical analyses were conducted using IBM SPSS Statistics 25.0 software (SPSS Inc., Chicago, USA) for Windows. The significance level considered was *p* < 0.05.

Descriptive analyses of the sample’s age, level of education, and sex were performed (Table 1).

**TABLE 1:** Sample sociodemographics.

		*n*	%	Mean	SD
Age				66.72	4.32
Total IQ				103.98	8.76
Total NEUROPSI				101.80	7.36
Quality of life (Q-LES-Q)				76.68	9.84
Sex					
	Female	58	65.9		
	Male	30	34.1		
Education level					
	Junior high school	4	4.5		
	High school	21	23.9		
	College/university	25	28.4		
	Postgraduate	38	43.2		
Socioeconomic status					
	Lower-middle (C−)	1	1.1		
	Middle-middle (C)	2	2.3		
	Upper-middle (C +)	13	14.8		
	Upper (A/B)	72	81.8		

IQ, Intelligence Quotient; Q-LES-Q, Quality of Life Enjoyment and Satisfaction Questionnaire; SD, Standard Deviation.

Multiple linear regression analyses were performed using the three dynamic proxy measures of CR as predictors: verbal intelligence (vocabulary, WAIS-III-R), leisure activities (hobbies), and physical activities (total physical index, YPAS).

Before running the regression analyses, the linearity, homoscedasticity, independence and normality assumptions were checked, and correlations were run to assess whether age, level of education, or sex were associated with cognitive performance or resting-state EEG variables.

A series of multiple linear regression models were then performed with three index scores of the WAIS (POI, WMI, and PSI), the four cognitive processes of the NEUROPSI (attention, memory, language, and executive functions), and the six EEG components (beta, alpha1, alpha2, gamma, theta, and delta) as dependent variables. A Bonferroni correction was applied to the seven regression models of cognition (*p* < 0.05/7 = 0.007) and the six regression models of resting-state EEG (*p* < 0.05/6 = 0.008) to evaluate statistically significant models. Model 1 included verbal intelligence, physical, and leisure activities as predictors; then, age, sex, and level of education were entered all together as covariates in Model 2 to adjust for these variables.

## Results

Key sociodemographics for these participants are presented in Table 1.

We found correlations between some of the cognitive and resting-state EEG variables and age, sex, and level of education (Supplementary Table 2). Thus, these variables were adjusted in further linear regression models.

### Cognition

Multiple linear regression models for cognitive performance are summarized in Table 2 (WAIS results) and Table 3 (NEUROPSI results).

**TABLE 2:** Multiple linear regression analysis with WAIS-III-R indices as outcomes.

		Model 1^a^		Model 2^b^	
		β (95% CI)	*P*-value	β (95% CI)	*P*-value
POI	Verbal intelligence	3.734 (2.078, 5.390)	<0.001*	2.955 (1.275, 4.635)	0.001*
	Physical activities	0.055 (−0.059, 0.170)	0.337	0.028 (−0.078, 0.135)	0.600
	Leisure activities	0.013 (−0.098, 0.124)	0.820	0.062 (−0.044, 0.169)	0.247
	Age	−		−1.160 (−1.737, −0.583)	<0.001
	Sex	−		0.978 (−4.193, 6.148)	0.708
	Level of education	−		3.438 (0.540, 6.335)	0.021
	a. *R*^2^ = 0.217, *F*(3, 84) = 7.739, *p* = < 0.001 b. *R*^2^ = 0.376, *F*(6, 81) = 8.141, *p* = < 0.001*				
WMI	Verbal intelligence	1.329 (0.702, 1.955)	<0.001*	1.384 (0.690, 2.078)	<0.001*
	Physical activities	−0.025 (−0.068, 0.018)	0.256	−0.020 (−0.064, 0.025)	0.380
	Leisure activities	−0.036 (−0.078, 0.006)	0.094	−0.045 (−0.089, −0.002)	0.043
	Age	−		0.211 (−0.028, 0.449)	0.083
	Sex	−		0.640 (−1.495, 2.776)	0.552
	Level of education	−		−0.268 (−1.465, 0.929)	0.657
	a. *R*^2^ = 0.187, *F*(3, 84) = 6.461, *p* = 0.001 b. *R*^2^ = 0.228, *F*(6, 81) = 3.980, *p* = 0.002*				
PSI	Verbal intelligence	3.907 (1.374, 6.441)	0.003*	4.221 (1.892, 6.550)	0.001*
	Physical activities	0.209 (0.034, 0.383)	0.020*	0.126 (−0.022, 0.274)	0.093
	Leisure activities	−0.045 (−0.215, 0.124)	0.598	0.087 (−0.060, 0.234)	0.244
	Age	−		−2.456 (−3.256, −1.656)	<0.001
	Sex	−		−1.612 (−8.781, 5.558)	0.656
	Level of education	−		−0.531 (−4.549, 3.487)	0.793
	a: *R*^2^ = 0.164, *F*(3, 84) = 5.476, *p* = 0.002 b: *R*^2^ = 0.453, *F*(6, 81) = 11.175, *p* = < 0.001*				

POI, Perceptual Organization Index; WMI, Working Memory Index; PSI, Processing Speed Index; CI, confidence interval. *Significant at the Bonferroni corrected alpha level of 0.007.

**TABLE 3:** Multiple linear regression analysis with NEUROPSI cognitive processes as outcomes.

		Model 1^a^		Model 2^b^	
		B (95% CI)	*P*-value	B (95% CI)	*P*-value
Attention	Verbal intelligence	0.226 (0.070, 0.381)	0.005	0.255 (0.080, 0.430)	0.005
	Physical activities	−0.003 (−0.014, 0.007)	0.538	−0.004 (−0.015, 0.007)	0.493
	Leisure activities	0.001 (−0.009, 0.012)	0.809	0.002 (−0.009, 0.013)	0.721
	Age	−		−0.007 (−0.067, 0.053)	0.818
	Sex	−		−0.112 (−0.652, 0.427)	0.679
	Level of education	−		−0.115 (−0.418, 0.187)	0.451
	a: *R*^2^ = 0.312, *F*(3, 84) = 3.025, *p* = 0.034 b: *R*^2^ = 0.333, *F*(6, 81) = 1.685, *p* = 0.135				
Memory	Verbal intelligence	0.422 (0.147, 0.697)	0.003*	0.384 (0.088, 0.679)	0.012
	Physical activities	−0.006 (−0.025, 0.013)	0.558	−0.001 (−0.020, 0.018)	0.900
	Leisure activities	0.021 (0.003, 0.040)	0.023*	0.016 (−0.002, 0.035)	0.085
	Age	−		0.070 (−0.032, 0.171)	0.175
	Sex	−		−1.367 (−2.276, −0.458)	0.004
	Level of education	−		0.099 (−0.411, 0.608)	0.701
	a: *R*^2^ = 0.176, *F*(3, 84) = 5.982, *p* = 0.001 b: *R*^2^ = 0.263, *F*(6, 81) = 4.819, *p* = < 0.001*				
Language	Verbal intelligence	0.255 (0.048, 0.462)	0.017*	0.248 (0.016, 0.480)	0.036
	Physical activities	0.004 (−0.010, 0.019)	0.537	0.006 (−0.009, 0.020)	0.451
	Leisure activities	0.011 (−0.003, 0.025)	0.109	0.009 (−0.006, 0.024)	0.218
	Age	−		0.043 (−0.037, 0.122)	0.291
	Sex	−		0.262 (−0.452, 0.975)	0.468
	Level of education	−		0.018 (−0.382, 0.418)	0.931
	a: *R*^2^ = 0.344, *F*(3, 84) = 3.763, *p* = 0.014 b: *R*^2^ = 0.379, *F*(6, 81) = 2.268, *p* = 0.045*				
Executive functions	Verbal intelligence	0.557 (0.307, 0.806)	<0.001*	0.573 (0.302, 0.845)	<0.001*
	Physical activities	−0.003 (−0.021, 0.014)	0.699	0.000 (−0.017, 0.018)	0.955
	Leisure activities	−0.003 (−0.020, 0.014)	0.712	−0.009 (−0.027, 0.008)	0.278
	Age	−		0.125 (0.032, 0.218)	0.009
	Sex	−		−0.040 (−0.875, 0.795)	0.924
	Level of education	−		−0.105 (−0.573, 0.363)	0.658
	a: *R*^2^ = 0.437, *F*(3, 84) = 6.624, *p* = < 0.001 b: *R*^2^ = 0.509, *F*(6, 81) = 4.733, *p* = < 0.001*				

CI, confidence interval. *Significant at the Bonferroni corrected alpha level of 0.007.

For the WAIS indices, a significant regression equation was found for the POI [*F*(6, 81) = 8.141, *p* < 0.001], with an *R*^2^ of 0.376, and verbal intelligence was a positive predictor [β = 2.955, 95% CI (1.275, 4.635), *p* = 0.001]. Another significant regression was found for the WMI [*F*(6, 81) = 3.980, *p* = 0.002], with an *R*^2^ of 0.228, and verbal intelligence as a positive predictor [β = 1.384, 95% CI (0.690, 2.078), *p* < 0.001]. The PSI also showed a significant regression [*F*(6, 81) = 11.175, *p* < 0.001], with an *R*^2^ of 0.453, and verbal intelligence was a positive predictor [β = 4.221, 95% CI (1.892, 6.550), *p* = 0.001].

The NEUROPSI results only showed a significant regression for the Executive functions regression [*F*(6, 81) = 4.733, *p* < 0.001], with an *R*^2^ of 0.509, and verbal intelligence as a positive predictor [β = 0.573, 95% CI (0.302, 0.845), *p* < 0.001].

All statistically significant models (Bonferroni corrected: *p* < 0.007) were found even after adjusting for age, sex, and level of education.

**FIGURE 1:**
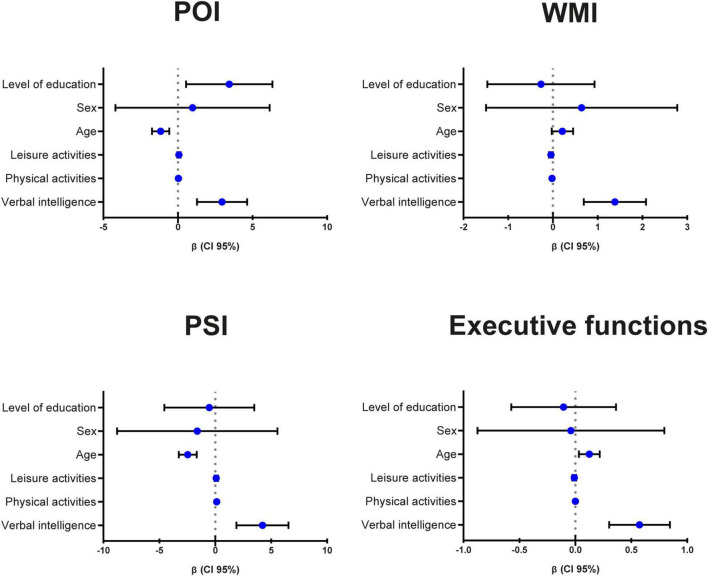
Visual summary of the statistically significant (Bonferroni corrected: *p* < 0.007) multiple linear regressions for cognitive performance. POI, Perceptual Organization Index; WMI, Working Memory Index; PSI, Processing Speed Index; CI, confidence interval.

A visual summary of the statistically significant (Bonferroni corrected: *p* < 0.007) multiple linear regressions for cognitive performance is presented in Figure 1.

### Electroencephalogram

Multiple linear regression models for EEG are summarized in Table 4. A significant regression equation was found for alpha2 [*F*(6, 81) = 3.488, *p* = 0.004], with an *R*^2^ of 0.453, with leisure activities as a positive predictor [β = 0.019, 95% CI (0.009, 0.028), *p* < 0.001]. Additionally, delta [*F*(6, 81) = 2.816, *p* = 0.015] showed a significant regression with an *R*^2^ of 0.415, and verbal intelligence was a positive predictor [β = 0.241, 95% CI (0.092, 0.390), *p* = 0.002].

**TABLE 4:** Multiple linear regression analysis with resting-state EEG components as outcomes.

		Model 1^a^		Model 2^b^	
		B (95% CI)	*P*-value	B (95% CI)	*P*-value
Beta	Verbal intelligence	−0.060 (−0.202, 0.081)	0.398	−0.127 (−0.281, 0.026)	0.103
	Physical activities	−0.004 (−0.014, 0.006)	0.396	−0.003 (−0.013, 0.007)	0.552
	Leisure activities	0.007 (−0.003, 0.016)	0.170	0.006 (−0.004, 0.016)	0.236
	Age	−		−0.011 (−0.064, 0.041)	0.666
	Sex	−		−0.556 (−1.028, −0.083)	0.022
	Level of education	−		0.254 (−0.011, 0.519)	0.060
	a: *R*^2^ = 0.187, *F*(3, 84) = 1.010, *p* = 0.392 b: *R*^2^ = 0.347, *F*(6, 81) = 1.853, *p* = 0.099				
Alpha1	Verbal intelligence	−0.032 (−0.170, 0.105)	0.641	−0.119 (−0.268, 0.030)	0.116
	Physical activities	0.009 (0.000, 0.019)	0.060	0.010 (0.001, 0.020)	0.036
	Leisure activities	0.010 (0.000, 0.019)	0.040*	0.009 (−0.001, 0.018)	0.072
	Age	−		−0.005 (−0.057, 0.046)	0.839
	Sex	−		0.015 (−0.445, 0.474)	0.949
	Level of education	−		0.344 (0.86, 0.601)	0.010
	a: *R*^2^ = 0.300, *F*(3, 84) = 2.760, *p* = 0.047 b: *R*^2^ = 0.412, *F*(6, 81) = 2.764, *p* = 0.017*				
Alpha2	Verbal intelligence	−0.085 (−0.220, 0.051)	0.216	−0.020 (−0.166, 0.126)	0.783
	Physical activities	−0.004 (−0.014, 0.005)	0.358	−0.007 (−0.017, 0.002)	0.119
	Leisure activities	0.015 (0.006, 0.024)	0.002*	0.019 (0.009, 0.028)	<0.001*
	Age	−		−0.055 (−0.106, −0.005)	0.031
	Sex	−		0.226 (−0.223, 0.676)	0.319
	Level of education	−		−0.231 (−0.483, 0.020)	0.071
	a: *R*^2^ = 0.348, *F*(3, 84) = 3.854, *p* = 0.012 b: *R*^2^ = 0.453, *F*(6, 81) = 3.488, *p* = 0.004*				
Gamma	Verbal intelligence	−0.104 (−0.245, 0.038)	0.148	−0.097 (−0.258, 0.063)	0.232
	Physical activities	−0.004 (−0.013, 0.006)	0.469	−0.004 (−0.014, 0.006)	0.469
	Leisure activities	−0.001 (−0.011, 0.008)	0.824	−0.001 (−0.011, 0.009)	0.840
	Age	−		0.003 (−0.052, 0.058)	0.912
	Sex	−		0.132 (−0.362, 0.626)	0.597
	Level of education	−		−0.025 (−0.302, 0.252)	0.859
	a: *R*^2^ = 0.190, *F*(3, 84) = 1.053, *p* = 0.374 b: *R*^2^ = 0.201, *F*(6, 81) = 0.567, *p* = 0.755				
Theta	Verbal intelligence	−0.020 (−0.164, 0.124)	0.782	−0.011 (−0.172, 0.150)	0.892
	Physical activities	0.001 (−0.009, 0.011)	0.836	0.002 (−0.008, 0.012)	0.672
	Leisure activities	0.002 (−0.008, 0.011)	0.738	0.000 (−0.010, 0.010)	0.980
	Age	−		0.027 (−0.028, 0.082)	0.334
	Sex	−		−0.348 (−0.843, 0.147)	0.165
	Level of education	−		−0.051 (−0.329, 0.226)	0.714
	a: *R*^2^ = 0.049, *F*(3, 84) = 0.067, *p* = 0.977 b: *R*^2^ = 0.192, *F*(6, 81) = 0.519, *p* = 0.793				
Delta	Verbal intelligence	0.193 (0.056, 0.330)	0.006*	0.241 (0.092, 0.390)	0.002*
	Physical activities	−0.005 (−0.014, 0.005)	0.325	−0.004 (−0.013, 0.006)	0.434
	Leisure activities	0.000 (−0.009, 0.010)	0.918	−0.001 (−0.010, 0.008)	0.823
	Age			0.039 (−0.012, 0.090)	0.133
	Sex			−0.345 (−0.804, 0.113)	0.138
	Level of education			−0.209 (−0.466, 0.048)	0.110
	a: *R*^2^ = 0.307, *F*(3, 84) = 2.916, *p* = 0.039 b: *R*^2^ = 0.415, *F*(6, 81) = 2.816, *p* = 0.015				

CI, confidence interval. *Significant at the Bonferroni corrected alpha level of 0.008.

All statistically significant models (Bonferroni corrected: *p* < 0.008) were found even after adjusting for age, sex, and level of education.

**FIGURE 2:**
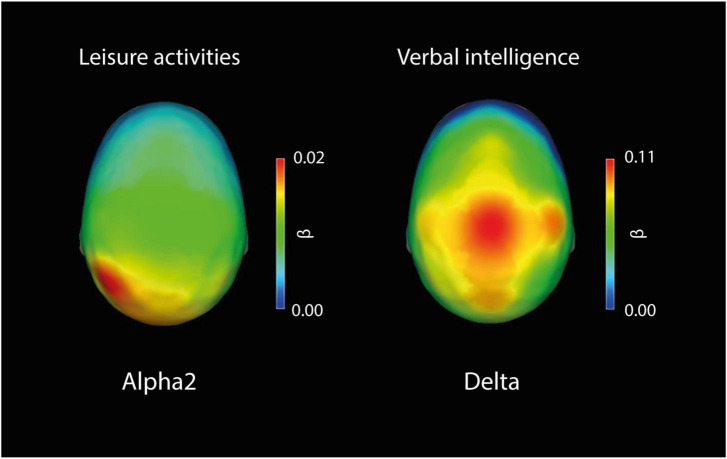
Topographic representation of the scalp distribution of beta values for each electrode of the frequency bands with its statistically significant (Bonferroni corrected: *p* < 0.008) predictors. Leisure activities as a predictor of Alpha2: All electrodes were significant. Verbal intelligence as a predictor of delta: C3, C4, P3, P4, O2, Fz, Cz, and Pz were significant.

A topographic representation of the scalp distribution of beta values for each electrode of the frequency bands with its statistically significant (Bonferroni corrected: *p* < 0.008) predictors is presented in Figure 2.

## Discussion

The aim of the study was to evaluate the association of CR with cognition and resting-state EEG in healthy older adults using dynamic proxy measures of CR.

Our first hypothesis was that dynamic proxy measures of CR would relate to fluid cognitive abilities, as reported in previous studies (Malek-Ahmadi et al., 2017; Serra et al., 2019). We found that not only fluid cognitive abilities but also crystallized abilities seem to be influenced by dynamic proxy measures of CR. Verbal intelligence was a positive predictor of perceptual organization, working memory, processing speed, and executive functions. As already reported by Boyle et al. (2021), verbal intelligence is a more robust cross-sectional measure of CR in comparison to education, occupational complexity, leisure activities, and exercise. These results are in line with the findings of the meta-analysis performed by Opdebeeck et al. (2016), where CR was positively associated with language, memory, working memory, executive function, visuospatial abilities, and general cognition. Even though the proxies of CR used were different between the studies, the results were similar. However, this result contrasts with the study of Ritchie et al. (2013) because they used years of education as a proxy of CR, which is a static proxy, and did not find any association with processing speed. The dynamic proxy measures seem to better reflect the fluid process of building CR over the lifespan (Malek-Ahmadi et al., 2017).

However, contrary to expectations, leisure and physical activities were not predictors of cognitive performance. Similar results were found by Fleck et al. (2019), who reported that the exercise factor group displayed no effect on cognition. The absence of associations between leisure and physical activities and cognition may be due to the lack of standardization of these variables, such as the frequency, intensity, duration, and type of activities, as concluded by some authors in previous studies (Wang et al., 2012; Anatürk et al., 2021).

Our second hypothesis was that dynamic proxy measures of CR would associate positively with alpha power and negatively with theta power of the resting-state EEG. Our findings support the hypothesis that a higher dynamic proxy measure is related to higher alpha power but do not support their relationship with lower theta activity. We found that leisure activities were a significant positive predictor of alpha2, particularly in posterior regions, which agrees with previous studies even using different proxies: static, dynamic, or a combination of both (Fleck et al., 2017, 2019; Sanchez-Lopez et al., 2018; Babiloni et al., 2020). This result may be due to compensatory processes. It has been demonstrated that the reduction in alpha rhythms in aging is related to a gradual loss of cholinergic function (Babiloni et al., 2020). Robertson (2013) proposed a model regarding the role of the noradrenergic system in mediating CR (represented by enrichment/mental stimulation), which leads to a set of brain mechanisms (disease compensation or modification) that reduce the risk of AD. One of these brain mechanisms is cholinergic rescue, which may explain the higher posterior alpha observed with higher scores of leisure activities. It is important to highlight that higher alpha has been related to better cognitive performance in older adults (Roca-Stappung et al., 2012; Barry and De Blasio, 2017; Choi et al., 2019; Kamal et al., 2020; Zangrossi et al., 2021).

An unexpected result was verbal intelligence as a positive predictor of delta, particularly in central regions. In the aging population, generalized EEG slowing characterized by increased power in theta and delta frequency bands, which are also related to cognitive decline, has been reported (Barry and De Blasio, 2017; Choi et al., 2019; Kamal et al., 2020; Kumral et al., 2020). Therefore, we were expecting a protective effect by CR proxies, a reduction of slower frequency bands, and our result seemed counterintuitive. Verbal intelligence was associated not only with higher AP in the delta but also with better cognitive performance. The different brain mechanisms underlying CR may explain this finding; perhaps verbal intelligence acts as the reserve theory effect of neural efficiency, i.e., less use of neural resources despite displaying better cognitive performance (Cabeza et al., 2018). In some cases, CR can even mask a cognitive decline process mediating the association between the pathological features of aging and cognitive performance (Arenaza-Urquijo et al., 2015; Gorges et al., 2017). Another possible explanation may be that individuals with higher verbal intelligence have a better structure of alpha rhythm, with a modulated fusiform amplitude; this modulation may change alpha spindles into an enveloping of slow frequency in the delta range (Chang et al., 2011). However, these heterogeneous results in the elderly population are expected because they are related to the aging process and its less distinct and more random brain functioning (Zangrossi et al., 2021).

Additionally, physical activities were not a predictor of either cognitive performance or resting-state EEG. As we already mentioned, Fleck et al. (2019) did not find an effect of the exercise factor on cognition or resting-state EEG connectivity, and Landau et al. (2012) reported a lack of association of physical activities with an indicator of β-amyloid deposition (carbon 11–labeled Pittsburgh Compound B). Similarly, in a longitudinal study, Verghese et al. (2003) concluded that leisure activities are a predictor of cognitive decline, whereas physical activities are not.

Some limitations of the present study are listed. First, the mean age of the sample was 66.72 (S.D. 4.32); however, old-old (over the age of 80) adults are a growing segment of the population that exhibit accelerated declines in cognitive function (Zhuravleva et al., 2014), and they were not included in our study. Additionally, our sample’s level of education and socioeconomic status does not represent the aging Mexican population. Although the inclusion criteria were the completion of junior high school and the absence of socioeconomic disadvantages, the sample recruited was particularly highly educated (*M* = 15.75 years of education, SD = 3.91) and from an upper socioeconomic status (82% of the sample). Even though the level of education was adjusted in the regression models, both the higher educational level and socioeconomic status of our sample may bias the conclusions. Thus, the findings are less generalizable and have a narrow scope.

The third limitation is that we performed a cross-sectional study, so the conclusions about the associations between CR and the behavioral and brain responses lack causality. For instance, it has been proposed that older adults who implement fewer leisure activities may be in a prodromal phase of AD (Verghese et al., 2003). Another limitation is the overlap of the CR proxies. The hobby score included an item about physical activities, and some leisure activities imply a physical activation (traveling and shopping). However, the interpretations can be integrated into the dynamic proxy measures of CR. Future studies should consider older samples or perform longitudinal studies to assess the relationship of these variables in the long term to better understand the evolution of CR’s underlying brain mechanisms and cognitive trajectories.

## Conclusion

In conclusion, the dynamic proxy measures of CR seem to relate to resting-state EEG and cognitive performance differently. These findings suggest that implementing leisure activities and tasks to increase vocabulary not just as prevention strategies but even as interventions in later life may promote better cognitive performance through compensation or neural efficiency mechanisms.

## Data availability statement

The dataset analyzed for this study can be found in Figshare: Cognitive Reserve, Resting EEG and Cognition: https://doi.org/10.6084/m9.figshare.19313822.

## Ethics statement

The Ethics Committee of the Institute of Neurobiology at the National Autonomous University of Mexico approved this project (INEU/SA/CB/109, protocol 030-H-RM), which followed the Ethical Principles for Medical Research Involving Human Subjects established by the Declaration of Helsinki. All participants signed informed consent forms.

## Author contributions

MF-D, RB-C, JS-P, and TF designed the study and planned the analyses. TF collected the data and cleaned the EEG data. RB-C processed the EEG data. MF-D conducted the analyses and prepared the first draft. MF-D and JS-P prepared all tables and figures. RB-C, JS-P, TF, CG-P, and MR-C provided feedback and comments on all versions of the manuscript. All authors contributed to the article and read and approved the submitted version.

## Supplementary material

Supplementary Figure 1 Topographic representation of the first six components of the EEG: Component 1 (Beta), Component 2 (Alpha1), Component 3 (Alpha2), Component 4 (Gamma), Component 5 (Theta), and Component 6 (Delta).
