# Supplementary material for: Verbal intelligence and leisure activities are associated with cognitive performance and resting-state electroencephalogram
Source: Front Aging Neurosci. 2022 Oct 4;14:921518. doi: 10.3389/fnagi.2022.921518 (PMC9577299; doi:10.3389/fnagi.2022.921518)
Supplement: Supplementary file 1 [file Table_1.docx]

**Supplementary Table 1.**

*Rotated component matrix*

| **Variables** | **Components** | | | | | | | | | | |
| --- | --- | --- | --- | --- | --- | --- | --- | --- | --- | --- | --- |
|  | **1** | **2** | **3** | **4** | **5** | **6** | **7** | **8** | **9** | **10** | **11** |
| P4-beta2 | **0.879** | 0.230 | 0.222 | 0.139 |  | 0.133 |  |  |  |  |  |
| Pz-beta2 | **0.869** | 0.277 | 0.222 | 0.187 |  | 0.120 |  |  |  |  |  |
| P3-beta2 | **0.860** | 0.231 | 0.240 | 0.195 |  | 0.143 |  | 0.167 |  |  |  |
| C4-beta2 | **0.851** | 0.315 | 0.179 | 0.244 | 0.150 | 0.101 |  |  |  |  |  |
| Cz-beta2 | **0.840** | 0.349 | 0.164 | 0.257 | 0.105 |  | 0.156 |  |  |  |  |
| C3-beta2 | **0.819** | 0.298 | 0.235 | 0.296 | 0.140 |  |  |  |  |  |  |
| Fz-beta2 | **0.808** | 0.423 | 0.172 | 0.261 | 0.110 |  | 0.110 |  |  |  |  |
| F3-beta2 | **0.745** | 0.334 | 0.251 | 0.348 | 0.109 |  | 0.138 |  |  | 0.115 |  |
| O2-beta2 | **0.714** | 0.329 | 0.358 | 0.243 |  | 0.200 |  |  | 0.157 |  |  |
| T6-beta2 | **0.709** | 0.295 | 0.256 | 0.238 |  | 0.239 |  |  | 0.189 |  |  |
| C4-beta1 | **0.706** | 0.328 | 0.301 | 0.135 | 0.289 | 0.191 | 0.118 |  | 0.318 |  |  |
| F4-beta2 | **0.700** | 0.336 | 0.110 | 0.356 | 0.128 |  |  |  |  | 0.396 |  |
| Cz-beta3 | **0.695** | 0.210 | 0.214 | 0.554 |  |  |  |  |  |  |  |
| Pz-beta3 | **0.683** | 0.104 | 0.309 | 0.572 |  | 0.158 | 0.107 |  |  |  |  |
| O1-beta2 | **0.674** | 0.326 | 0.327 | 0.299 |  | 0.157 | 0.208 | 0.304 | 0.136 |  |  |
| P4-beta3 | **0.659** |  | 0.297 | 0.588 |  | 0.177 |  | 0.147 |  |  |  |
| Cz-beta1 | **0.657** | 0.378 | 0.286 | 0.140 | 0.260 | 0.134 | 0.228 |  | 0.362 |  |  |
| C3-beta1 | **0.652** | 0.319 | 0.379 | 0.174 | 0.254 | 0.163 | 0.112 |  | 0.322 |  |  |
| P3-beta1 | **0.647** | 0.265 | 0.386 |  | 0.308 | 0.195 |  | 0.104 | 0.396 |  |  |
| Fz-beta1 | **0.646** | 0.443 | 0.284 | 0.140 | 0.293 |  | 0.165 |  | 0.314 |  |  |
| Fz-beta3 | **0.639** | 0.224 | 0.220 | 0.584 |  |  | 0.216 |  |  |  |  |
| C4-beta3 | **0.638** | 0.195 | 0.173 | 0.621 |  | 0.148 | 0.160 |  |  |  |  |
| P3-beta3 | **0.624** |  | 0.283 | 0.564 |  | 0.132 |  | 0.364 | -0.111 |  |  |
| F3-beta1 | **0.620** | 0.370 | 0.311 | 0.201 | 0.270 | 0.147 | 0.157 |  | 0.363 |  |  |
| P4-beta1 | **0.614** | 0.260 | 0.375 |  | 0.362 | 0.197 |  |  | 0.424 |  |  |
| Pz-beta1 | **0.611** | 0.326 | 0.365 |  | 0.290 | 0.184 | 0.166 |  | 0.432 |  |  |
| F4-beta1 | **0.602** | 0.363 | 0.215 | 0.211 | 0.319 |  | 0.109 |  | 0.323 | 0.281 |  |
| T6-beta1 | **0.532** | 0.318 | 0.326 |  | 0.232 | 0.231 |  |  | 0.522 |  |  |
| Fp2-beta1 | **0.527** | 0.347 | 0.187 | 0.217 | 0.264 |  | 0.346 |  | 0.273 | 0.428 |  |
| F8-beta2 | **0.505** | 0.399 |  | 0.482 |  | 0.134 |  |  | 0.312 | 0.163 |  |
| F8-beta1 | **0.484** | 0.439 | 0.124 | 0.252 | 0.264 | 0.177 | 0.181 |  | 0.475 |  |  |
| O1-beta1 | **0.464** | 0.236 | 0.375 |  | 0.352 | 0.190 | 0.233 | 0.167 | 0.457 |  |  |
| Pz-alfa1 | 0.183 | **0.869** | 0.157 |  | 0.193 | 0.149 |  |  |  |  |  |
| T6-alfa1 | 0.265 | **0.860** | 0.237 |  | 0.209 | 0.114 |  |  | 0.164 |  |  |
| P4-alfa1 | 0.218 | **0.859** | 0.226 |  | 0.207 | 0.192 |  |  |  |  |  |
| Cz-alfa1 | 0.315 | **0.854** | 0.211 |  | 0.196 | 0.111 | 0.146 |  |  |  |  |
| F8-alfa1 | 0.320 | **0.843** | 0.176 |  | 0.181 |  | 0.185 |  | 0.118 |  |  |
| P3-alfa1 | 0.139 | **0.841** | 0.299 |  | 0.243 | 0.191 |  |  |  |  |  |
| C4-alfa1 | 0.314 | **0.840** | 0.248 |  | 0.209 | 0.175 | 0.103 |  |  |  |  |
| Fz-alfa1 | 0.337 | **0.836** | 0.222 |  | 0.229 |  | 0.106 |  |  |  |  |
| F4-alfa1 | 0.353 | **0.832** | 0.235 |  | 0.220 |  | 0.114 |  |  |  |  |
| O2-alfa1 |  | **0.830** | 0.250 |  | 0.210 | 0.150 |  |  | 0.121 |  |  |
| F7-alfa1 | 0.375 | **0.827** | 0.231 |  | 0.202 |  | 0.138 |  |  |  |  |
| Fp2-alfa1 | 0.338 | **0.825** | 0.189 |  | 0.224 |  | 0.210 |  |  |  |  |
| F3-alfa1 | 0.372 | **0.825** | 0.246 |  | 0.221 |  | 0.111 |  |  |  |  |
| C3-alfa1 | 0.329 | **0.825** | 0.313 |  | 0.223 | 0.144 |  |  |  |  |  |
| Fp1-alfa1 | 0.348 | **0.823** | 0.189 |  | 0.230 |  | 0.225 |  |  |  |  |
| T4-alfa1 | 0.243 | **0.792** | 0.223 |  | 0.127 | 0.154 |  |  | 0.261 |  |  |
| O1-alfa1 |  | **0.779** | 0.221 |  | 0.203 | 0.127 |  |  |  |  |  |
| T3-alfa1 | 0.246 | **0.767** | 0.339 |  | 0.183 | 0.121 |  |  | 0.124 |  |  |
| T5-alfa1 | 0.134 | **0.748** | 0.410 |  | 0.280 | 0.155 |  |  | 0.171 |  |  |
| P3-alfa2 | 0.187 | 0.156 | **0.896** |  | 0.169 | 0.208 |  |  |  |  |  |
| C3-alfa2 | 0.240 | 0.253 | **0.880** | 0.159 | 0.144 | 0.139 |  |  |  |  |  |
| Cz-alfa2 | 0.226 | 0.283 | **0.861** | 0.171 | 0.162 |  |  |  |  |  |  |
| F3-alfa2 | 0.276 | 0.271 | **0.850** | 0.138 | 0.212 | 0.125 |  |  |  |  |  |
| T5-alfa2 | 0.106 |  | **0.845** |  | 0.183 | 0.260 |  | 0.123 | 0.136 |  |  |
| C4-alfa2 | 0.223 | 0.317 | **0.844** | 0.182 | 0.186 | 0.168 |  |  |  |  |  |
| P4-alfa2 | 0.248 | 0.209 | **0.842** |  | 0.199 | 0.206 |  |  |  |  |  |
| F4-alfa2 | 0.218 | 0.287 | **0.836** | 0.137 | 0.263 | 0.103 |  |  |  |  |  |
| Pz-alfa2 | 0.300 | 0.214 | **0.827** |  | 0.164 | 0.148 |  |  | -0.154 |  |  |
| T3-alfa2 | 0.203 | 0.286 | **0.824** |  |  | 0.152 |  |  |  |  |  |
| Fz-alfa2 | 0.231 | 0.304 | **0.824** | 0.113 | 0.255 |  |  |  |  |  |  |
| O2-alfa2 | 0.128 | 0.156 | **0.819** |  | 0.116 | 0.269 |  |  | 0.202 |  |  |
| F7-alfa2 | 0.301 | 0.265 | **0.818** | 0.167 | 0.169 | 0.136 | 0.153 |  | 0.124 |  |  |
| Fp1-alfa2 | 0.259 | 0.275 | **0.814** |  | 0.233 | 0.153 | 0.124 |  |  |  |  |
| Fp2-alfa2 | 0.230 | 0.299 | **0.803** |  | 0.255 | 0.155 |  |  |  | 0.127 |  |
| O1-alfa2 |  |  | **0.784** |  | 0.196 | 0.297 |  |  | 0.158 |  |  |
| T6-alfa2 | 0.220 | 0.252 | **0.778** |  | 0.125 | 0.200 |  |  | 0.311 |  |  |
| F8-alfa2 | 0.240 | 0.400 | **0.753** | 0.133 | 0.235 | 0.213 |  |  | 0.189 |  |  |
| T4-alfa2 | 0.217 | 0.413 | **0.718** | 0.164 |  | 0.216 |  |  | 0.230 |  | 0.124 |
| Pz-gamma | 0.266 |  | 0.139 | **0.880** |  |  | 0.199 | 0.124 |  |  |  |
| P4-gamma | 0.228 |  | 0.102 | **0.864** |  |  | 0.172 | 0.299 |  |  |  |
| Cz-gamma | 0.258 |  | 0.199 | **0.861** |  | 0.128 | 0.214 |  |  |  |  |
| C3-gamma | 0.135 |  |  | **0.835** |  |  | 0.136 |  |  |  |  |
| C4-gamma | 0.202 |  |  | **0.817** |  |  | 0.394 |  |  |  |  |
| F8-gamma | 0.104 |  |  | **0.812** |  |  |  |  | 0.179 | 0.149 |  |
| Fz-gamma | 0.212 |  | 0.266 | **0.769** |  | 0.101 | 0.416 |  |  |  |  |
| F8-beta3 | 0.303 | 0.120 |  | **0.725** |  | 0.124 |  |  | 0.183 | 0.236 |  |
| P3-gamma | 0.149 |  |  | **0.722** |  |  | 0.173 | 0.603 |  |  |  |
| F3-gamma | 0.150 |  | 0.191 | **0.706** |  |  | 0.336 |  |  | 0.145 |  |
| T6-gamma | 0.116 |  | 0.145 | **0.689** |  | 0.180 |  | 0.102 |  |  |  |
| O2-beta3 | 0.384 |  | 0.309 | **0.679** |  |  | 0.151 | 0.125 |  |  | 0.126 |
| T4-gamma |  |  |  | **0.677** |  | 0.129 |  | 0.513 |  |  |  |
| T3-gamma |  | -0.115 | -0.146 | **0.668** | 0.115 | 0.113 |  | 0.438 |  |  |  |
| O2-gamma |  |  |  | **0.656** |  |  | 0.190 | 0.155 |  |  | 0.100 |
| C3-beta3 | 0.631 | 0.188 | 0.220 | **0.645** |  | 0.118 | 0.119 |  |  |  |  |
| T4-beta3 | 0.237 | 0.109 |  | **0.642** |  | 0.219 |  | 0.419 | 0.167 |  |  |
| T3-beta3 | 0.121 |  |  | **0.637** | 0.142 | 0.122 |  | 0.497 |  |  |  |
| T6-beta3 | 0.348 |  | 0.224 | **0.624** |  | 0.250 |  |  |  |  |  |
| F4-gamma | 0.144 |  |  | **0.622** |  |  |  |  |  | 0.602 |  |
| F3-beta3 | 0.440 |  | 0.318 | **0.615** |  |  | 0.306 |  |  | 0.123 |  |
| F4-beta3 | 0.452 | 0.135 |  | **0.580** |  |  |  |  |  | 0.526 |  |
| O1-beta3 | 0.288 |  | 0.203 | **0.579** |  |  | 0.531 | 0.406 |  |  |  |
| T3-beta2 | 0.353 |  |  | **0.467** | 0.181 | 0.221 |  | 0.324 | 0.286 |  |  |
| P3-theta | 0.116 |  | 0.186 |  | **0.886** | 0.219 |  |  |  |  | -0.164 |
| T5-theta |  | 0.107 | 0.154 |  | **0.885** | 0.224 |  |  | 0.109 |  |  |
| P4-theta |  | 0.174 | 0.189 |  | **0.870** | 0.240 |  |  |  |  | -0.139 |
| Pz-theta | 0.137 | 0.243 | 0.200 |  | **0.866** | 0.277 |  |  |  |  |  |
| C4-theta | 0.162 | 0.177 | 0.236 |  | **0.859** | 0.303 |  |  |  |  |  |
| C3-theta | 0.181 | 0.160 | 0.261 |  | **0.856** | 0.322 |  |  |  |  |  |
| Cz-theta | 0.166 | 0.236 | 0.209 |  | **0.834** | 0.324 |  |  |  |  |  |
| O1-theta |  | 0.299 | 0.166 |  | **0.816** | 0.294 |  |  |  |  |  |
| T6-theta | 0.161 | 0.434 | 0.141 |  | **0.786** | 0.253 |  |  | 0.146 |  |  |
| F4-theta | 0.163 | 0.319 | 0.255 |  | **0.748** | 0.298 |  |  |  |  | 0.165 |
| O2-theta | 0.103 | 0.421 | 0.181 |  | **0.742** | 0.319 |  |  | 0.133 |  |  |
| Fz-theta | 0.136 | 0.344 | 0.240 |  | **0.741** | 0.251 |  |  |  |  | 0.153 |
| F7-theta |  | 0.325 | 0.189 |  | **0.732** | 0.237 |  |  |  |  | 0.263 |
| Fp1-theta |  | 0.294 | 0.125 |  | **0.731** | 0.226 |  |  |  |  | 0.440 |
| F3-theta | 0.160 | 0.300 | 0.264 |  | **0.731** | 0.315 |  |  |  |  | 0.188 |
| Fp2-theta |  | 0.364 | 0.141 |  | **0.724** | 0.253 | 0.104 |  |  |  | 0.386 |
| T3-theta |  | 0.196 | 0.190 |  | **0.695** | 0.261 |  |  | 0.125 |  | 0.132 |
| F8-theta | 0.134 | 0.484 | 0.130 |  | **0.672** | 0.307 |  |  | 0.103 |  | 0.224 |
| T4-theta | 0.137 | 0.471 | 0.220 |  | **0.636** | 0.356 |  |  | 0.158 |  | 0.134 |
| Cz-delta | 0.134 |  | 0.202 | 0.145 | 0.236 | **0.889** |  |  |  |  |  |
| P3-delta | 0.175 |  | 0.179 |  | 0.269 | **0.884** |  |  |  |  |  |
| C4-delta | 0.146 | 0.103 | 0.206 | 0.122 | 0.264 | **0.874** |  |  |  |  |  |
| C3-delta | 0.149 |  | 0.177 |  | 0.290 | **0.873** |  |  |  |  |  |
| Pz-delta | 0.157 | 0.103 | 0.213 |  | 0.263 | **0.872** |  |  |  |  |  |
| O2-delta | 0.103 | 0.153 | 0.205 |  | 0.264 | **0.845** |  |  |  |  |  |
| T6-delta | 0.113 | 0.270 | 0.156 | 0.129 | 0.258 | **0.828** |  |  | 0.135 |  |  |
| Fz-delta | 0.131 | 0.183 | 0.148 | 0.120 | 0.317 | **0.808** |  |  |  |  |  |
| F3-delta | 0.153 | 0.162 | 0.215 | 0.155 | 0.268 | **0.805** |  |  |  |  | 0.117 |
| F4-delta | 0.151 | 0.192 | 0.192 | 0.123 | 0.362 | **0.770** |  |  |  |  | 0.165 |
| T4-delta |  | 0.297 | 0.185 | 0.197 | 0.157 | **0.749** |  |  |  |  | 0.113 |
| T5-delta |  | 0.193 | 0.124 |  | 0.275 | **0.732** |  |  |  |  |  |
| T3-delta | 0.122 |  | 0.253 | 0.225 | 0.208 | **0.720** |  |  | 0.153 |  | 0.142 |
| O1-delta |  |  |  | -0.109 | 0.179 | **0.660** |  |  |  |  |  |
| F7-delta |  | 0.218 | 0.189 | 0.166 | 0.359 | **0.614** |  |  |  |  | 0.279 |
| P4-delta |  |  | 0.238 |  | 0.564 | **0.571** |  |  |  |  | -0.261 |
| F8-delta |  | 0.354 | 0.163 | 0.147 | 0.349 | **0.506** |  |  |  |  | 0.401 |
| F7-beta3 |  | 0.114 |  | 0.227 |  |  | **0.944** |  |  |  |  |
| F7-gamma |  |  | -0.101 | 0.297 |  |  | **0.920** |  |  |  |  |
| Fp1-beta3 | 0.147 |  | 0.104 | 0.290 |  |  | **0.911** |  |  | 0.101 |  |
| Fp1-gamma |  |  |  | 0.327 |  |  | **0.907** |  |  | 0.150 |  |
| F7-beta2 | 0.359 | 0.249 |  | 0.280 |  |  | **0.823** |  | 0.102 |  |  |
| Fp1-beta2 | 0.458 | 0.238 | 0.202 | 0.355 |  |  | **0.706** |  |  | 0.173 |  |
| O1-gamma |  |  |  | 0.526 |  |  | **0.651** | 0.393 |  |  |  |
| F7-beta1 | 0.436 | 0.313 | 0.147 | 0.230 | 0.199 | 0.107 | **0.611** |  | 0.362 |  |  |
| Fp1-beta1 | 0.507 | 0.310 | 0.264 | 0.227 | 0.229 |  | **0.561** |  | 0.275 | 0.128 |  |
| T5-beta3 |  |  |  | 0.263 |  |  |  | **0.952** |  |  |  |
| T5-gamma |  |  |  | 0.275 |  |  |  | **0.944** |  |  |  |
| T5-beta2 | 0.240 |  |  | 0.204 |  |  |  | **0.924** |  |  |  |
| T5-beta1 | 0.350 |  | 0.230 |  | 0.188 | 0.134 |  | **0.783** | 0.302 |  |  |
| T4-beta2 | 0.405 | 0.396 |  | 0.328 | 0.104 | 0.183 |  | **0.429** | 0.373 | 0.101 |  |
| T4-beta1 | 0.424 | 0.483 | 0.197 |  | 0.195 | 0.229 |  | 0.206 | **0.540** |  |  |
| T3-beta1 | 0.408 | 0.160 | 0.255 | 0.181 | 0.219 | 0.274 |  | 0.179 | **0.536** |  |  |
| O2-beta1 | 0.490 | 0.317 | 0.387 |  | 0.266 | 0.212 |  |  | **0.534** |  |  |
| Fp2-gamma | 0.106 |  |  | 0.473 |  | -0.119 | 0.418 |  |  | **0.695** |  |
| Fp2-beta3 | 0.231 |  |  | 0.391 |  | -0.112 | 0.524 |  |  | **0.637** |  |
| Fp2-beta2 | 0.504 | 0.252 | 0.119 | 0.315 |  |  | 0.350 |  |  | **0.624** |  |
| Fp1-delta |  |  |  |  | 0.358 | 0.426 |  |  |  |  | **0.695** |
| Fp2-delta |  |  |  |  | 0.193 | 0.453 |  |  |  |  | **0.648** |
| Eigenvalue | 67.92 | 21.68 | 11.47 | 8.24 | 6.07 | 4.90 | 4.60 | 3.81 | 2.69 | 2.07 | 1.91 |
| Variation explained, %* | 14.46 | 13.67 | 12.39 | 11.65 | 10.89 | 9.41 | 5.48 | 3.86 | 2.99 | 1.85 | 1.43 |
| Cumulative variances, %* | 14.46 | 28.13 | 40.53 | 52.18 | 63.07 | 72.49 | 77.97 | 81.38 | 84.83 | 86.68 | 88.11 |

*Note.* Extraction method: Principal component analysis, Rotation method: Varimax with Kaiser normalization.

*Rotation sum of squared loadings.
