# Supplementary material for: Verbal intelligence and leisure activities are associated with cognitive performance and resting-state electroencephalogram
Source: Front Aging Neurosci. 2022 Oct 4;14:921518. doi: 10.3389/fnagi.2022.921518 (PMC9577299; doi:10.3389/fnagi.2022.921518)
Supplement: Supplementary file 2 [file Table_2.docx]

**Supplementary Table 2.**

*Correlations between cognitive performance and resting-state EEG with sociodemographic variables*

|  | **Age** | **Sex** | **Level of education** |
| --- | --- | --- | --- |
|  | **r (p-value)** | **r (p-value)** | **r (p-value)** |
| **POI** | -0.289 (0.006)* | 0.000 (0.997) | 0.354 (0.001)* |
| **WMI** | 0.177 (0.100) | 0.089 (0.411) | 0.157 (0.145) |
| **PSI** | -0.538 (<0.001)* | -0.179 (0.095) | 0.025 (0.817) |
| **Attention** | -0.009 (0.932) | -0.090 (0.405) | 0.049 (0.649) |
| **Memory** | 0.148 (0.168) | -0.278 (0.009)* | 0.140 (0.192) |
| **Language** | 0.183 (0.087) | 0.107 (0.320) | 0.180 (0.093) |
| **Executive functions** | 0.267 (0.012)* | 0.039 (0.721) | 0.175 (0.103) |
| **Beta** | -0.036 (0.736) | -0.220 (0.039)* | 0.098 (0.363) |
| **Alpha1** | 0.044 (0.687) | 0.093 (0.391) | 0.259 (0.015)* |
| **Alpha2** | -0.110 (0.310) | -0.027 (0.802) | -0.162 (0.133) |
| **Gamma** | 0.020 (0.851) | 0.059 (0.583) | -0.069 (0.521) |
| **Theta** | 0.062 (0.568) | -0.148 (0.169) | -0.079 (0.464) |
| **Delta** | 0.126 (0.241) | -0.185 (0.084) | -0.046 (0.672) |

*Note.* POI: Perceptual Organization Index; WMI: Working Memory Index; PSI: Processing Speed Index.

* p < 0.05
