# Supplementary figures and images for: Verbal intelligence and leisure activities are associated with cognitive performance and resting-state electroencephalogram
Source: Front Aging Neurosci. 2022 Oct 4;14:921518. doi: 10.3389/fnagi.2022.921518 (PMC9577299; doi:10.3389/fnagi.2022.921518)

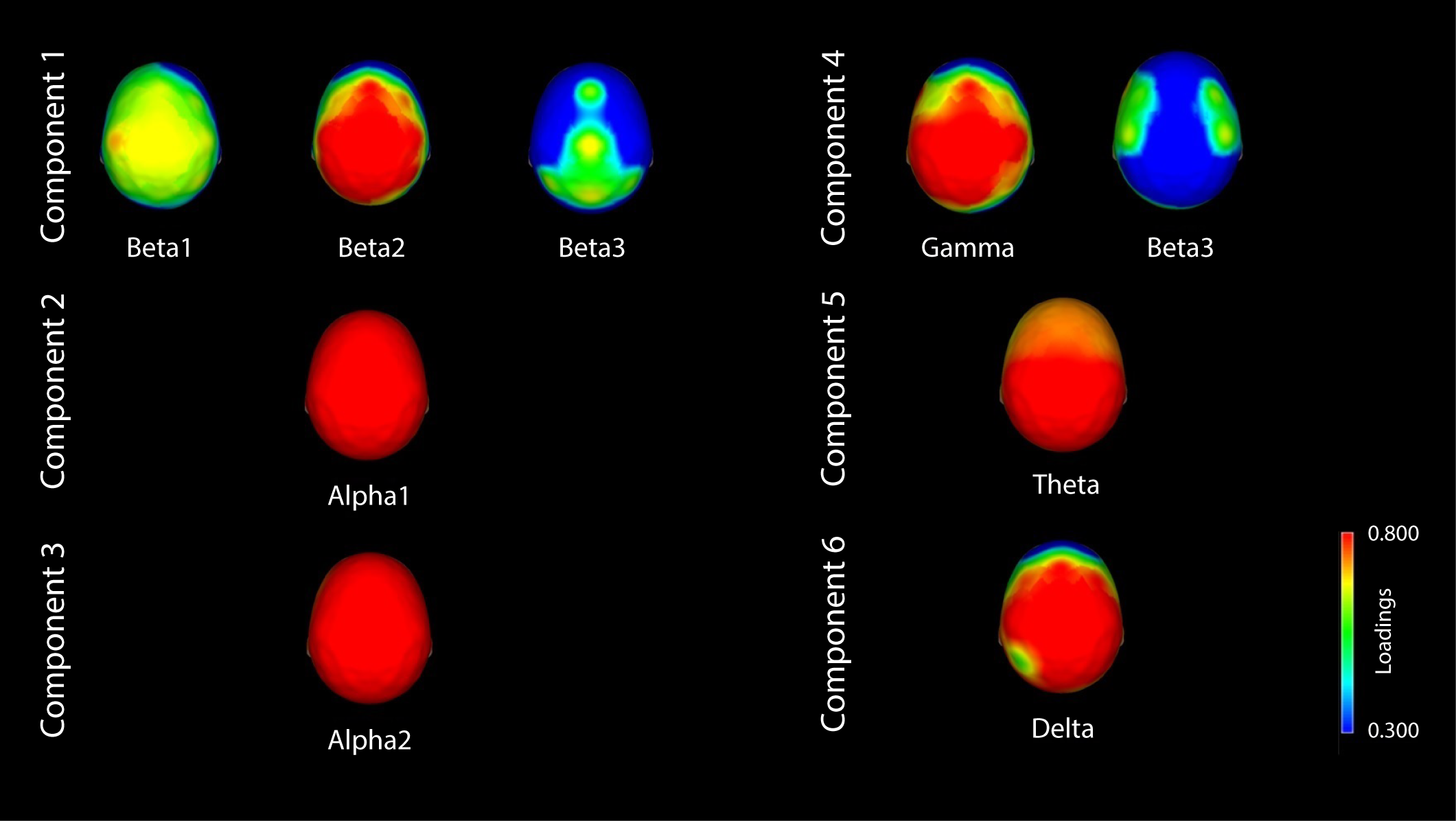

Supplement: Supplementary Figure 1 — Topographic representation of the first six components of the EEG: Component 1 (Beta), Component 2 (Alpha1), Component 3 (Alpha2), Component 4 (Gamma), Component 5 (Theta), and Component 6 (Delta). [file Image_1.TIF]
